# Exploring the Acceptability and Feasibility of Remote Blood Pressure Measurements and Cognition Assessments Among Participants Recruited From a Safety-Net Emergency Department (Reach Out Cognition): Nonrandomized Mobile Health Trial

**DOI:** 10.2196/54010

**Published:** 2024-05-28

**Authors:** Mackenzie Dinh, Chun Chieh Lin, Candace Whitfield, Zahera Farhan, William J Meurer, Sarah Bailey, Lesli E Skolarus

**Affiliations:** 1 Department of Emergency Medicine University of Michigan Ann Arbor, MI United States; 2 Division of Health Services Research Department of Neurology The Ohio State University Columbus, OH United States; 3 Bridges into the Future Flint, MI United States; 4 Davee Department of Neurology Northwestern University Chicago, IL United States

**Keywords:** hypertension, cognition, mobile health, Bluetooth, remote, monitoring, monitor, low income, mHealth, hypertensive, cardiology, cardiovascular, feasibility, acceptability, satisfaction, RCT, randomized controlled trial, assessment, blood pressure, neurological, mobile health

## Abstract

**Background:**

Hypertension is a prevalent cardiovascular risk factor disproportionately affecting Black Americans, who also experience a higher incidence of Alzheimer disease and Alzheimer disease–related dementias. Monitoring blood pressure (BP) and cognition may be important strategies in reducing these disparities.

**Objective:**

The objective of the Reach Out Cognition study was to explore the feasibility and acceptability of remote cognitive and BP assessments in a predominantly Black, low-income population.

**Methods:**

Reach Out was a randomized, controlled, mobile health–based clinical trial to reduce BP among patients with hypertension at an emergency department in a safety-net hospital (ie, a US hospital in which 25% of the patients are Medicaid recipients). Upon conclusion of Reach Out, participants were given the option of continuing into an extension phase, Reach Out Cognition, that included Bluetooth-enabled BP monitoring and digital cognitive assessments for 6 months. Digital cognitive assessments were text message–linked online surveys of the Self-Administered Gerocognitive Exam and Quality of Life in Neurological Disorders scale. BP assessments were measured with Bluetooth-enabled BP cuffs paired with an app and the data were manually sent to the research team. Outcomes were feasibility (ie, enrollment and 3- and 6-month completion of digital cognitive and BP assessments) and acceptability of assessments using a 4-item validated survey, ranging from 1 (not acceptable) to 5 (completely acceptable).

**Results:**

Of the 211 Reach Out participants, 107 (50.7%) consented and 71 (33.6%) completed enrollment in Reach Out Cognition. Participants had a mean age of 49.9 years; 70.4% were female and 57.8% identified as Black. Among the 71 participants, 51 (72%) completed cognitive assessments at 3 months and 34 (48%) completed these assessments at 6 months. BP assessments were completed by 37 (52%) and 20 (28%) of the 71 participants at 3 and 6 months, respectively. Participants were neutral on the acceptability of the digital cognitive assessments (mean 3.7) and Bluetooth self-measured BP (SMBP) monitoring (mean 3.9). Participants noted challenges syncing the BP cuff to the app, internet connection, and transmitting the data to the research team.

**Conclusions:**

Enrollment and assessment completion were low, while acceptability was moderate. Technological advances will eliminate some of the Bluetooth SMBP barriers and offer new strategies for cognitive assessments. Subsequent studies could benefit from offering more comprehensive support to overcome Bluetooth-related hurdles, such as personalized training materials, video conferencing, or in-person research team support. Alternatively, strategies that do not require pairing with an app and passive transmission of data could be considered. Overall, further research is warranted to optimize participant engagement and overcome technological challenges.

**Trial Registration:**

ClinicalTrials.gov NCT03422718; https://clinicaltrials.gov/study/NCT03422718

## Introduction

In the United States, Black people experience the highest incidence of Alzheimer disease and Alzheimer disease–related dementias, with a 2-fold greater incidence compared to that of the non-Hispanic White population [1-5]. Numerous large epidemiological studies have demonstrated an association between elevated blood pressure (BP) and the development of mild cognitive impairment and dementia [6,7]. Considering the disproportionate burden of hypertension among Black people and individuals with low income, interventions that specifically prioritize these high-risk groups are needed.

Mobile health (mHealth) is defined by the World Health Organization as a medical or public health practice that is supported by mobile devices such as smartphones or tablets [8]. Black Americans have higher smartphone usage than their White counterparts [9]. Thus, mHealth may offer an approach to overcome racial disparities by providing a new way to promote health and complement health care [10]. Within mHealth, there is growing interest in mHealth cognitive assessments, with ongoing studies aiming to develop cognitive self-monitoring apps and expand their implementation in clinical trials [11]. Bluetooth-enabled BP cuffs offer advantages such as automatic transfer of readings to smartphone apps, graphical displays, and gamification to increase engagement. While small-scale studies have shown promise, the effectiveness of Bluetooth-enabled BP cuffs in larger, more diverse populations still needs to be tested [12].

In this context, we conducted Reach Out Cognition, a pilot nonrandomized trial of Bluetooth-enabled BP monitoring and digital cognitive assessments in patients with hypertension who were recruited from a safety-net emergency department (ED), defined as hospitals in the United States where over 25% of patients are Medicaid recipients [13], and elected to continue in an extension study. Specifically, we sought to evaluate the feasibility and acceptability of Bluetooth-enabled BP monitoring and digital cognitive assessments to inform the design of future trials using these measures as part of the intervention or outcomes.

## Methods

### Study Design

Reach Out Cognition was an extension of the Reach Out trial. Briefly, the Reach Out trial was a randomized, controlled, 2×2×2 factorial-design mHealth clinical trial to reduce BP among safety-net ED patients with hypertension who were seeking care for conditions with a high likelihood of discharge from the ED (ClinicalTrials.gov identifier NCT03422718) [14]. Specifically, patients were eligible if (1) they had at least one documented systolic BP reading ≥160 mm Hg or diastolic BP reading ≥100 mm Hg; (2) after achieving criterion 1, at least one repeat BP measurement remained ≥140 mm Hg (systolic) or ≥90 mm Hg (diastolic); (3) they were likely to be discharged from the ED; and (4) they possessed a cell phone with text messaging capability. We excluded patients who were critically ill, otherwise unable to give informed consent, non-English–speaking, incarcerated, pregnant, had a preexisting condition that made 1-year follow-up unlikely, currently used 3 or more antihypertensive agents (suggesting resistant hypertension), or had dementia/cognitive impairment. Enrollment occurred from March 2019 to March 2020.

Participants were randomized to the following groups: (1) prompted self-measured BP (SMBP) monitoring, (2) tailored healthy behavior text messaging, and (3) facilitated primary care provider scheduling and transportation. Overall, systolic BP declined after 6 months (median mean difference −9.2 mm Hg, 95% CI −12.2 to −6.3) and 12 months (−6.6 mm Hg, 95% CI −9.3 to −3.8), without a difference across the 8 treatment arms [15]. There was no difference in systolic BP among the 3 mHealth components. After completion of Reach Out, participants were given the opportunity to continue in Reach Out Cognition, where they would continue to receive daily healthy behavior text messages and weekly prompted BP self-monitoring. Additional study details have been published elsewhere [16].

### Reach Out Cognition Study Population

The study was conducted from September 2020 to February 2022. Adults were eligible if they were no more than 6 months from completion of their 12-month Reach Out outcomes. Reach Out Cognition was designed with the goal of inclusivity and generalizability, thus allowing for inclusion of all cell phones with the capability to send picture text messages. We excluded participants with baseline mild cognitive impairment according to a score <8 on the telephone-Montreal Cognitive Assessment (T-MoCA-Short) [17].

### Enrollment Procedures

**Figure 1:**
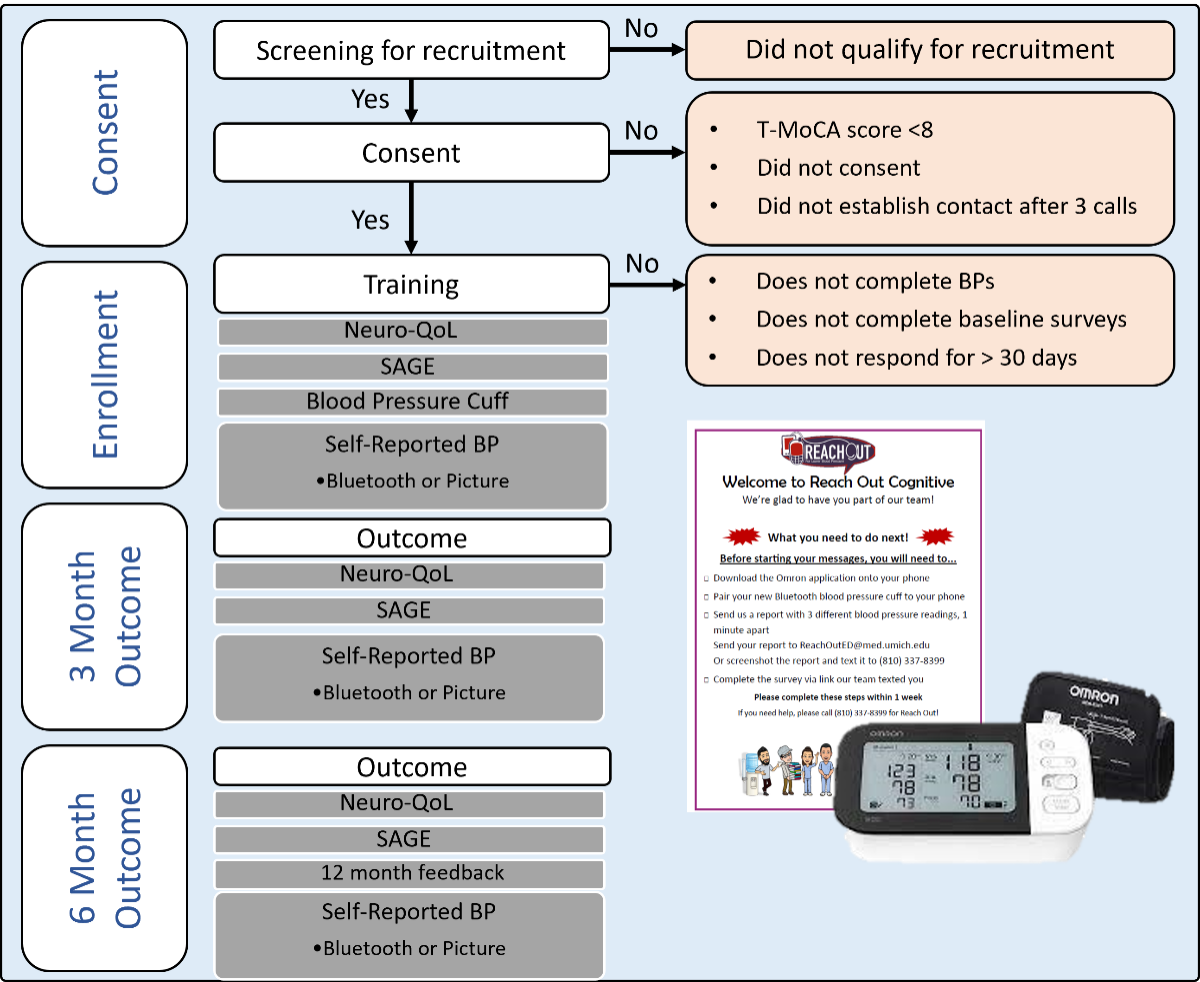
Study flow and outcomes. BP: blood pressure; Neuro-QoL: Quality of Life in Neurological Disorders; SAGE: Self-Administered Gerocognitive Exam; T-MoCA: telephone-Montreal Cognitive Assessment.

Eligible Reach Out participants were contacted by the study team and invited to participate in Reach Out Cognition. The study design is described in Figure 1. Those interested were consented and then mailed a wireless, Bluetooth-enabled OMRON 7 series upper-arm BP monitor (BP7350) or an OMRON 7 series wrist BP monitor (BP6350) for those needing a larger cuff. Participants were also mailed written instructions for taking their BP and using the Bluetooth BP monitor, along with instructions for completing cognitive outcomes based on their phone’s capability, an individualized MoCA feedback form, and a checklist of enrollment elements. Due to COVID-19, all aspects of training were done remotely through phone, video, written materials, or online trainings [18]. Participants with smartphone capability were assisted in downloading the OMRON app associated with the Bluetooth-compatible BP monitor. Participants received optional assistance from the study team for training on their specific OMRON cuff, OMRON app, and web-based surveys.

During enrollment, participants were texted a link to an online Qualtrics survey that contained baseline cognitive assessments, including a modified Self-Administered Gerocognitive Exam (SAGE) and Quality of Life in Neurological Disorders (Neuro-QoL)–Cognition scale [19,20]. Participants who opted not to take the cognitive assessments through Qualtrics were sent paper copies. Each week, participants were texted a personalized message with instructions specifying what remained incomplete (ie, BP, cognitive assessment) for enrollment. To facilitate enrollment, multiple steps were undertaken. First, participants were called and texted once weekly for 1 month for assistance (maximum of 4 follow-up texts and 4 follow-up calls). Phone or video calls with a study coordinator, dependent on the participant’s preference, were offered to help go through the Bluetooth connection step by step and to address any other barriers. Second, text messages included a link to the Reach Out website [18] to offer visual examples and training for completing enrollment elements. Third, each week, participants were texted a personalized message with instructions. Fourth, participants were also mailed phone-specific instructions, which included Bluetooth troubleshooting advice. If participants did not complete BP and cognitive assessments and had no contact with the study team for 1 month post enrollment, they were withdrawn from the study.

### mHealth Assessments

#### Data Collection

Outcome assessments were specific to whether the participant had a smartphone or feature phone (ie, a phone without the capability of downloading apps). Participants with smartphones were asked to email/text their BP data downloaded from their OMRON app to the study team. There was no connection between the OMRON app and the study team. Participants with feature phones were asked to send a photograph of their arm with the BP cuff on and 3 self-reported BP readings. The photograph was used to ensure the BP cuff was being worn appropriately. Cognitive assessments were completed through text message–linked online surveys administered through Qualtrics. Outcome assessments were conducted at 3 and 6 months (±6 weeks) to allow participants the opportunity to complete the assessments. BP assessment completion was defined as receipt of at least 3 BP measurements at 3 and 6 months. Cognitive assessment completion was defined as completion of the online assessment. Participants were contacted at least five times, with three text messages and two calls throughout the daytime, evening, and weekend hours to encourage assessment completion. If the participant expressed difficulty with self-measured digital cognitive assessments or BP measurements, they were contacted by the study team to provide assistance.

#### Outcomes

Acceptability and feasibility are key drivers for establishing clinical trial readiness and attaining desired clinical outcomes [21-24]. Feasibility was defined as the extent to which digital cognitive and BP assessments were completed [22]. Reach Out Cognition feasibility was defined as the proportion of eligible Reach Out participants who enrolled in Reach Out Cognition and the proportion of participants who completed the digital cognitive and BP assessments. Acceptability and satisfaction were measured with a survey texted to all participants. Acceptability of SMBP monitoring with the OMRON app and digital cognitive assessments was measured using a validated 4-item scale [22]. Questions related to the overall satisfaction with Reach Out Cognition and training, SMBP Bluetooth monitoring using an app, and self-administered and digital cognitive assessments. In addition, participants provided feedback on interactions with the study team, technology barriers, and overall experiences through Reach Out Cognition.

#### Exploratory Outcomes

Neuro-QoL and modified SAGE scores were compared at baseline, 3 months, and 6 months. Neuro-QoL–Cognitive short-form scores ranged from 1 to 40, with 40 indicating high cognitive function [20]. Modified SAGE scores ranged from 0 to 22, with a score of 17 and above considered normal cognition [19].

### Ethical Considerations

This study was approved by the University of Michigan Institutional Review Board (HUM00138470) and ED site Institutional Review Board (1199877). All participants provided informed consent at the time of enrollment. The original informed consent allowed primary data collection and for deidentified use in additional analyses and research studies. Participants of Reach Out Cognition were given an automated BP cuff at enrollment, US $20 after completion of the 3-month follow-up visit, and US $30 after completion of the 6-month follow-up visit.

### Statistical Analysis

Descriptive statistics were used to describe the study population and assess feasibility and acceptability. The self-reported measures were also assessed with descriptive statistics as continuous measures and dichotomized into agree (agree and completely agree vs neither agree nor disagree, disagree, and completely disagree). Cognitive measures were compared as continuous measures. Changes in these scores over the course of the study were assessed by comparing the mean scores at baseline, 3 months, and 6 months. The mean values of patient characteristics were compared with Student *t* tests. Estimated systolic BP was compared over time between groups using SAS Proc Mixed Procedures with the LSMEANS function to compare means with the Tukey honestly significant difference test.

## Results

### Participant Characteristics

**Figure 2:**
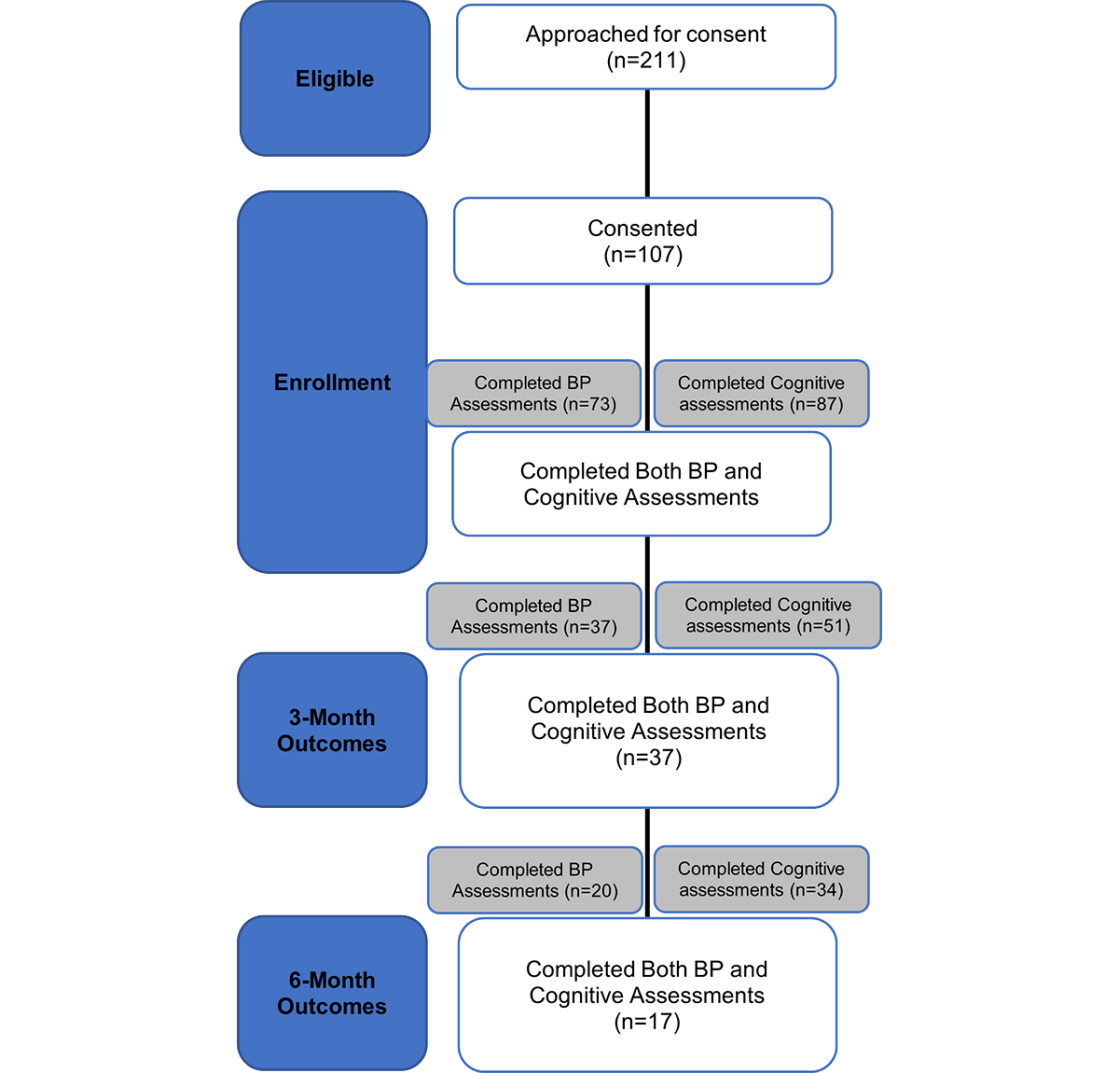
CONSORT (Consolidated Standards of Reporting Trials) diagram. BP: blood pressure.

A total of 211 participants completed Reach Out and were approached for Reach Out Cognition. Of those, 107 (50.7%) consented to Reach Out Cognition and 71 (33.6%) completed enrollment and were participants in Reach Out Cognition (Figure 2). The baseline characteristics of participants are shown in Table 1. Participants had a mean age of 49.9 (SD 11.3) years, 70.4% were female, and 57.8% identified as Black. Among the 71 participants, 27 (38%) reported having completed some high school, high school graduation, or trade school, while a larger proportion (62%) had achieved some college education or higher. Most participants reported having a primary care physician (94.4%), and 9.9% were uninsured. Overall, 83.1% of the participants had a previous diagnosis of hypertension and 66.2% were taking BP medications. Lack of transportation was cited as a barrier to health care access by 21.1% of the participants. The mean T-MoCA-Short total score was 9.62 (SD 1.5).

**Table 1:** Baseline participant characteristics (N=71).

Characteristics		Participants, n (%)
Female sex		50 (70.4)
Hispanic ethnicity		2 (2.8)
Black race		41 (57.8)
**Education**		
	Some HS^a^, HS graduate, or trade school	27 (38.0)
	Some college or higher	44 (62.0)
Married/living with someone		21 (29.6)
Not employed		37 (52.1)
Currently have a PCP^b^		67 (94.4)
Uninsured		7 (9.9)
Previous diagnosis of hypertension		59 (83.1)
**Comorbidities**		
	Diabetes	22 (31.0)
	Heart attack	6 (8.5)
	CHF^c^	7 (9.9)
	Kidney disease	5 (7.0)
	High cholesterol	27 (38.0)
	Cancer	5 (7.0)
	Stroke/TIA^d^	11 (15.5)
	Sleep apnea	28 (39.4)
	Dementia	1 (1.4)
	Lung disease	22 (31.0)
Prior medication for hypertension within the last 6 months		47 (66.2)
Lack of transportation		15 (21.1)
Downloaded OMRON app		71 (100.0)
**Phone model**		
	Android	31 (43.7)
	iPhone	32 (45.1)
	Google	2 (2.8)
	Other	6 (8.5)
**Phone use**		
	Text	71 (100.0)
	Picture messages	63 (88.7)
	Email	59 (83.1)
	Health/physical activity apps	31 (43.7)
	Social media apps	60 (84.5)
	Maps	57 (80.3)
	Other apps	17 (23.9)
**Access to the internet**		69 (97.2)
	Phone	52 (73.2)
	Personal computer	11 (15.5)
	Personal tablet/iPad	5 (7.0)
Completion of T-MoCA-Short^e^		69 (97.2)

^a^HS: high school.

^b^PCP: primary care physician.

^c^CHF: chronic heart failure.

^d^TIA: transient ischemic attack.

^e^Telephone-Montreal Cognitive Assessment short form.

### Technological Capability

Only 2.8% of participants had a nonsmartphone (feature phone). Most participants had an Android or iPhone smartphone. Participants used their phones for texting, picture messages, and email. Additionally, 43.7% of participants used health or physical activity apps on their phones, while social media apps were used by 84.5% of the participants (Table 1). Among the participants who reported health and physical activity app use, these apps were primarily reported in the use of fitness tracking, provider connection, and general health tracking (see Table S1 in Multimedia Appendix 1). Other apps were generally for entertainment, communication, or personalized use. Regarding internet access, 97.2% of participants reported having internet access. Among those with internet access, the majority used their phones to access the internet, followed by a personal computer, and a smaller proportion used a personal tablet/iPad (Table 1).

### Outcome Feasibility and Acceptability

Among the 71 participants, the digital cognitive assessments were completed by 51 participants (72%) at 3 months and by 34 participants (48%) at 6 months. At least one cognitive assessment was completed by 56 (79%) participants between the 3- and 6-month outcomes. The BP assessments (either through BP data downloaded or screenshot from their OMRON app, or a picture with self-reported BPs) were completed by 37 participants (52%) at 3 months and by 17 participants (28%) at 6 months. Among the 17 participants who completed 6-month outcomes, 71% (n=12) self-reported using the OMRON app, with 19% of these participants reporting that they manually entered their BPs into the app. Participants noted challenges syncing the BP cuff to the app, internet connection, and transmitting the data to the research team (eg, the research team did not have access to OMRON data). One participant noted, “just easier to look at the [BP] machine.”

Participants’ overall satisfaction with the study was assessed at 6 months. Among the 34 respondents, 79% (n=27) indicated being satisfied or very satisfied with the study. Participants were neutral regarding the acceptability of cognitive assessments and Bluetooth SMBP monitoring. The median scores of most items were in the neither agree nor disagree (3) to slightly agree (4) range (see Table S2 in Multimedia Appendix 1).

### Exploratory Outcomes

At baseline, the mean Neuro-QoL score was 31.5 (SD 6.5) and the mean modified SAGE score was 15.6 (SD 4.3). The Neuro-QoL scores were stable at 3 months (mean 31.5, SD 5.8) and 6 months (mean 33.6, SD 6.9). Similarly, the SAGE scores were stable from baseline after 3 months (mean 17.4, SD 4.7) and 6 months (mean 16.5, SD 3.0).

## Discussion

Reach Out Cognition evaluated the feasibility and acceptability of Bluetooth SMBP monitoring and digital cognitive assessments in a diverse population of adults with hypertension recruited from a safety-net ED who elected to enroll in an extended follow-up study. Overall, enrollment and outcome completion were low, whereas acceptability was moderate, suggesting that additional strategies are needed to encourage engagement of this population with Bluetooth SMBP monitoring and digital cognitive assessments.

The feasibility of remote assessments varied between cognitive and BP measurements. We found that more participants completed cognitive assessments than BP assessments. The cognitive assessments were completed via web-based surveys accessed from a text message link. While this is a preliminary strategy of assessment, as cognitive assessments are moving to apps, this may be a feasible approach that could complement apps.

In addition, the BP assessments required more steps than the cognitive assessments. This included pairing the Bluetooth-enabled BP cuff with the app and sending data from the app to the study team. These were noted as challenges by the participants. Some participants chose to manually enter BP readings into the app rather than through Bluetooth transmission. This may imply that the participants engaged in a way that aligns with their current routine and technological familiarity. Transmitting BP data from the app to the research or clinical team is now more readily available than when this pilot study was conducted [25]. Thus, technological advances will eliminate at least one of these barriers. Subsequent studies could benefit from offering more comprehensive support to overcome Bluetooth-related hurdles, such as personalized training materials, video conferencing, or in-person research team support. Alternatively, strategies that do not require pairing with an app and passive transmission of data could be considered.

This study has some limitations. First, Reach Out Cognition enrolled a selected population, given that it was an extension of a prior trial that used text messaging for BP monitoring for 12 months. This limits the generalizability of our findings. Because participants had already engaged in BP monitoring for 1 year through a different technique, feasibility may differ among people initiating BP measurements. Second, although participants used a BP cuff that synced to a mobile app, the researchers did not have direct access to the data within the mobile app. Instead, participants had to send their BP measurements to the research team via text message or other methods, creating an extra hurdle for participants in the study. Third, we did not perform a qualitative analysis after the study. Thus, little is known about the participants’ motivation to participate in the study. Finally, we cannot exclude the possibility that participants may have completed the BP measurements but did not transfer the data to the research team or that someone other than the participant completed the BP or cognitive assessments. However, this was advised against at the enrollment visit.

Overall, enrollment and digital cognitive and SMBP monitoring completion were low, while acceptability was moderate. As future interventions aim to harness the potential of mHealth for cognition, a mixed remote and in-person approach that allows personalization for participants based on their needs may be key for maximizing the feasibility and acceptability of these tools.

## Appendix

Multimedia Appendix 1 Participant app use (Table S1) and mobile health (mHealth) acceptability measures at 6-month follow-up (Table S2).

## Abbreviations

BP: blood pressure
ED: emergency department
mHealth: mobile health
Neuro-QoL: Quality of Life in Neurological Disorders
SAGE: Self-Administered Gerocognitive Exam
SMBP: self-measured blood pressure
T-MoCA-Short: telephone Montreal Cognitive Assessment short form

## Data Availability

The data sets generated during and/or analyzed during this study are available from the corresponding author on reasonable request.

## References

[1] Tang MX, Cross P, Andrews H, Jacobs DM, Small S, Bell K, Merchant C, Lantigua R, Costa R, Stern Y, Mayeux R (2001). Incidence of AD in African-Americans, Caribbean Hispanics, and Caucasians in northern Manhattan. Neurology.

[2] Mayeda ER, Glymour MM, Quesenberry CP, Whitmer RA (2016). Inequalities in dementia incidence between six racial and ethnic groups over 14 years. Alzheimers Dement.

[3] Plassman BL, Langa KM, Fisher GG, Heeringa SG, Weir DR, Ofstedal MB, Burke JR, Hurd MD, Potter GG, Rodgers WL, Steffens DC, McArdle JJ, Willis RJ, Wallace RB (2008). Prevalence of cognitive impairment without dementia in the United States. Ann Intern Med.

[4] Kuller LH, Lopez OL, Jagust WJ, Becker JT, DeKosky ST, Lyketsos C, Kawas C, Breitner JCS, Fitzpatrick A, Dulberg C (2005). Determinants of vascular dementia in the Cardiovascular Health Cognition Study. Neurology.

[5] Gurland BJ, Wilder DE, Lantigua R, Stern Y, Chen J, Killeffer EH, Mayeux R (1999). Rates of dementia in three ethnoracial groups. Int J Geriatr Psychiatry.

[6] Walker KA, Power MC, Gottesman RF (2017). Defining the relationship between hypertension, cognitive decline, and dementia: a review. Curr Hypertens Rep.

[7] Iadecola C, Yaffe K, Biller J, Bratzke LC, Faraci FM, Gorelick PB, Gulati M, Kamel H, Knopman DS, Launer LJ, Saczynski JS, Seshadri S, Zeki Al Hazzouri A, American Heart Association Council on Hypertension; Council on Clinical Cardiology; Council on Cardiovascular Disease in the Young; Council on Cardiovascular and Stroke Nursing; Council on Quality of Care and Outcomes Research; Stroke Council (2016). Impact of hypertension on cognitive function: a Scientific Statement from the American Heart Association. Hypertension.

[8] WHO Global Observatory for eHealth (2011). mHealth: New Horizons for Health through Mobile Technologies: Based on the Findings of the Second Global Survey on eHealth (Global Observatory for eHealth Series, Volume 3).

[9] Atske S, Perrin A (2021). Home broadband adoption, computer ownership vary by race, ethnicity in the U.S. Pew Research Center.

[10] Ray R, Sewell AA, Gilbert KL, Roberts JD (2017). Missed opportunity? Leveraging mobile technology to reduce racial health disparities. J Health Polit Policy Law.

[11] Thompson LI, Harrington KD, Roque N, Strenger J, Correia S, Jones RN, Salloway S, Sliwinski MJ (2022). A highly feasible, reliable, and fully remote protocol for mobile app-based cognitive assessment in cognitively healthy older adults. Alzheimers Dement.

[12] Rifkin DE, Abdelmalek JA, Miracle CM, Low C, Barsotti R, Rios P, Stepnowsky C, Agha Z (2013). Linking clinic and home: a randomized, controlled clinical effectiveness trial of real-time, wireless blood pressure monitoring for older patients with kidney disease and hypertension. Blood Press Monit.

[13] Burke G, Paradise J (2015). Safety-net emergency departments: a look at current experiences and challenges. KFF.

[14] Meurer WJ, Dinh M, Kidwell KM, Flood A, Champoux E, Whitfield C, Trimble D, Cowdery J, Borgialli D, Montas S, Cunningham R, Buis LR, Brown D, Skolarus L (2020). Reach out behavioral intervention for hypertension initiated in the emergency department connecting multiple health systems: study protocol for a randomized control trial. Trials.

[15] Skolarus LE, Dinh M, Kidwell KM, Lin CC, Buis LR, Brown DL, Oteng R, Giacalone M, Warden K, Trimble DE, Whitfield C, Farhan Z, Flood A, Borgialli D, Montas S, Jaggi M, Meurer WJ (2023). Reach out emergency department: a randomized factorial trial to determine the optimal mobile health components to reduce blood pressure. Circ Cardiovasc Qual Outcomes.

[16] Skolarus LE, Dinh M, Kidwell KM, Farhan Z, Whitfield C, Levine DA, Meurer WJ (2021). Supplement study update for Reach Out: a multi-arm randomized trial of behavioral interventions for hypertension initiated in the emergency department: Reach Out Cognition. Trials.

[17] Nasreddine ZS, Phillips NA, Bédirian V, Charbonneau S, Whitehead V, Collin I, Cummings JL, Chertkow H (2005). The Montreal Cognitive Assessment, MoCA: a brief screening tool for mild cognitive impairment. J Am Geriatr Soc.

[18] Reach Out.

[19] Scharre DW, Chang S, Murden RA, Lamb J, Beversdorf DQ, Kataki M, Nagaraja HN, Bornstein RA (2010). Self-administered Gerocognitive Examination (SAGE): a brief cognitive assessment Instrument for mild cognitive impairment (MCI) and early dementia. Alzheimer Dis Assoc Disord.

[20] Cella D, Lai JS, Nowinski CJ, Victorson D, Peterman A, Miller D, Bethoux F, Heinemann A, Rubin S, Cavazos JE, Reder AT, Sufit R, Simuni T, Holmes GL, Siderowf A, Wojna V, Bode R, McKinney N, Podrabsky T, Wortman K, Choi S, Gershon R, Rothrock N, Moy C (2012). Neuro-QOL. Neurology.

[21] Proctor E, Silmere H, Raghavan R, Hovmand P, Aarons G, Bunger A, Griffey R, Hensley M (2011). Outcomes for implementation research: conceptual distinctions, measurement challenges, and research agenda. Adm Policy Ment Health.

[22] Weiner BJ, Lewis CC, Stanick C, Powell BJ, Dorsey CN, Clary AS, Boynton MH, Halko H (2017). Psychometric assessment of three newly developed implementation outcome measures. Implement Sci.

[23] Bond C, Lancaster GA, Campbell M, Chan C, Eddy S, Hopewell S, Mellor K, Thabane L, Eldridge S (2023). Pilot and feasibility studies: extending the conceptual framework. Pilot Feasibility Stud.

[24] Lancaster GA, Dodd S, Williamson PR (2004). Design and analysis of pilot studies: recommendations for good practice. J Eval Clin Pract.

[25] Kaihara T, Intan-Goey V, Scherrenberg M, Falter M, Kario K, Akashi Y, Dendale P (2022). Automatic transmission of home blood pressure data can be effective in managing hypertension: a systematic review and meta-analysis. Eur Heart J Digit Health.

