# Supplementary material for: Exploring the Acceptability and Feasibility of Remote Blood Pressure Measurements and Cognition Assessments Among Participants Recruited From a Safety-Net Emergency Department (Reach Out Cognition): Nonrandomized Mobile Health Trial
Source: JMIR Form Res. 2024 May 28;8:e54010. doi: 10.2196/54010 (PMC11167316; doi:10.2196/54010)
Supplement: Multimedia Appendix 1 [file formative_v8i1e54010_app1.docx]

Table S1. Participant App Use.

| **Health/ Physical Activity Apps** | | | **Other Apps** | | |
| --- | --- | --- | --- | --- | --- |
| **Fitness Tracking** | **Provider Connection** | **Health Tracking** | **Entertainment** | **Communication** | **Personal** |
| - Lose it - Apple Watch App - Fitbit App - Step Counter - Blue Cross App - Nike Run Club - Apple Fitness - Planet Fitness App - Samsung Health - MyFitnessPal - Fit Step - Slimqueen - Step Counter - Achievement - Workout App: Healthy - Googlefit | - Hurley App - "Heart Dr. appt app" - Video Chat App for provider | - Sleep Monitoring App - Heart Healthy App - Blood Sugar App - Blood Pressure App - Smoking Cessation App - Diabetic App | - Games - Music - Podcasts - Books - Movies - Television - News Outlets - Shopping - Google | - Email - Zoom | - Work related Apps - Banking - Doorbell - Wallet - Hobbies |

Table S2. Reach Out Cognition Participant mHealth Acceptability Measures after 6 months^a^

|  | ***n* (%) Responding** | ***n* (%) Agree** | **mean(SD)** | **median (IQR)** |
| --- | --- | --- | --- | --- |
| **Cognitive Surveys** | | | | |
| *Cognitive surveys meets my approval.* | 33 (91.7) | 27 (75.0) | 4 (1.1) | 4 (4-5) |
| *Cognitive surveys are appealing to me.* | 33 (91.7) | 19 (52.8) | 3.7 (1.0) | 4 (3-4) |
| *I like the Cognitive surveys* | 33 (91.7) | 20 (55.6) | 3.7 (1.0) | 4 (3-4) |
| *I welcome the Cognitive surveys* | 31 (86.1) | 20 (55.6) | 3.8 (1.0) | 4 (3-4) |
| **Blood Pressure App** | | | | |
| *The blood pressure app connected to my blood pressure cuff meets my approval.* | 32 (88.9) | 23 (63.9) | 4 (0.9) | 4 (3-5) |
| *The blood pressure app connected to my blood pressure cuff is appealing to me.* | 32 (88.9) | 18 (50.0) | 3.7 (1.1) | 4 (3-5) |
| *I like the the blood pressure app connected to my blood pressure cuff* | 32 (88.9) | 25 (69.4) | 4 (0.9) | 4 (4-5) |
| *I welcome The blood pressure app connected to my blood pressure cuff* | 32 (88.9) | 24 (66.7) | 3.9 (0.8) | 4 (3.5-4.5) |

## ^a^0-5 completely disagree, disagree, neither agree nor disagree, agree, completely agree
